# Traumatic Spondylolisthesis of L5-S1 in a Pediatric Age: A Rarely Described Injury

**DOI:** 10.7759/cureus.69674

**Published:** 2024-09-18

**Authors:** Ana Esteves, David Gouveia, João Lixa, André Pinho, António Sousa

**Affiliations:** 1 Orthopaedics, Centro Hospitalar Tâmega e Sousa, Porto, PRT; 2 Orthopaedics, Centro Hospitalar São João, Porto, PRT

**Keywords:** lumbar spine injury, pediatric fractures, sacral frature, spinopelvic dissociation, spondylolisthesis in children

## Abstract

In this article, we aim to present the case of a five-year-old child with traumatic spondylolisthesis of L5-S1 associated with a sacral fracture, manifesting as a spinopelvic dissociation. The goal of surgical treatment is to reduce the deformity and provide posterior fixation to restore the sagittal alignment of the lumbosacral junction. One-year follow-up showed good clinical and radiological outcomes.

## Introduction

Traumatic spondylolisthesis of L5-S1 in a pediatric age is an extremely rare injury, typically associated with a high-energy mechanism. In traumatic spondylolisthesis, there is a flexion-distraction mechanism with fracture-dislocation of the L5-S1 facet joint complex associated with anterior slippage of L5. This injury is mostly found in multiple trauma patients and may be unrecognized in many cases [1].

In the presence of neurological deficits, conservative treatment will not promote adequate recovery. The best option to prevent progressive lumbosacral slippage and kyphosis and prevent neurological dysfunction is surgical realignment and stabilization of the lumbosacral junction [2].

Due to high-energy trauma, patients with L5-S1 traumatic spondylolisthesis often present with associated comorbidities such as thoracic and abdominal trauma, which may require urgent treatment. Due to the high mortality rate of these lesions, the actual number of lumbosacral dislocations may be underestimated [1]. To our knowledge, there are few reported cases.

## Case presentation

In this article, we describe the clinical case of a five-year-old healthy male child who was brought to the emergency department with an alleged history of a falling heavy object (cabinet weighing about 20 kilograms) over his spine, leading to a direct injury to the lumbar spine.

He was brought to the emergency department due to a cabinet falling on him, in the living room, resulting in direct trauma to the lumbar spine. The child had lumbosacral pain and was unable to walk due to pain. He had no other complaints and no neurological deficits.

**Figure 1:**
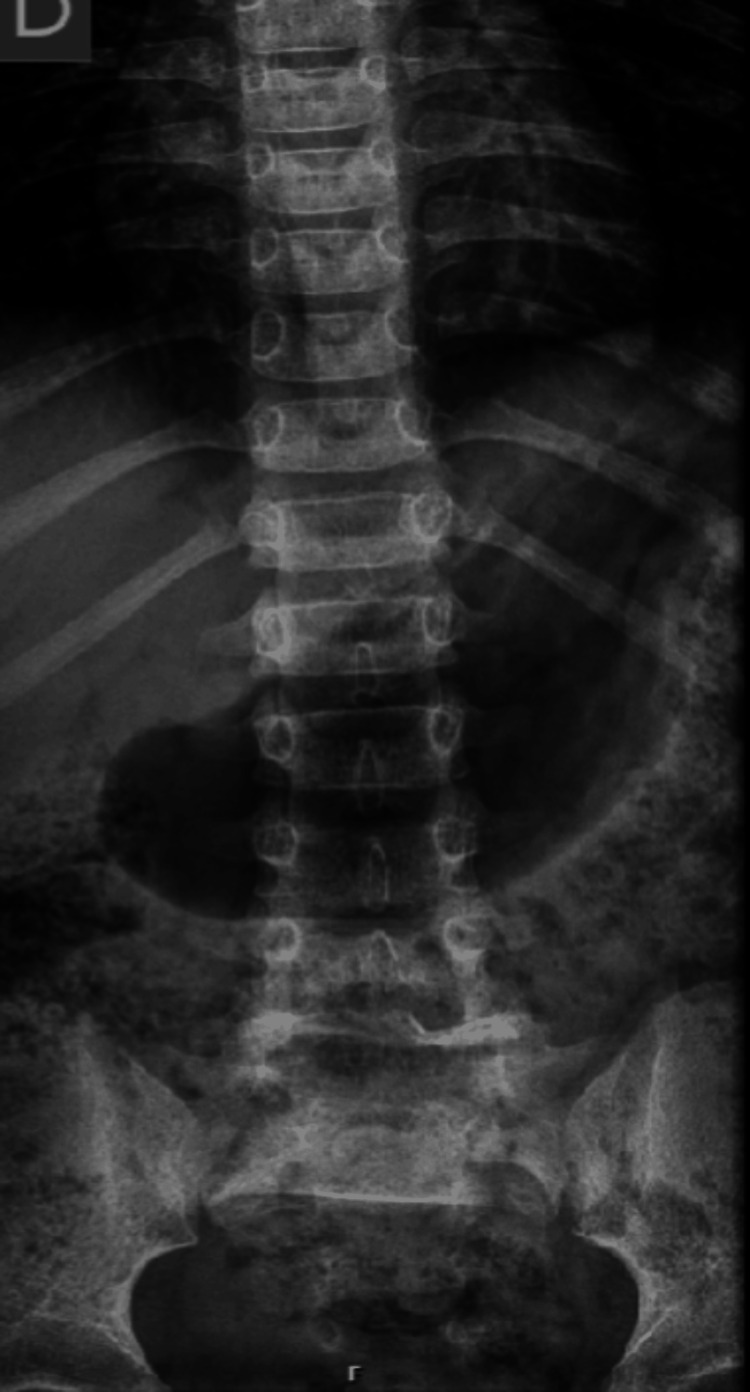
Radiograph showing suspected spondylolisthesis of L5-S1

**Figure 2:**
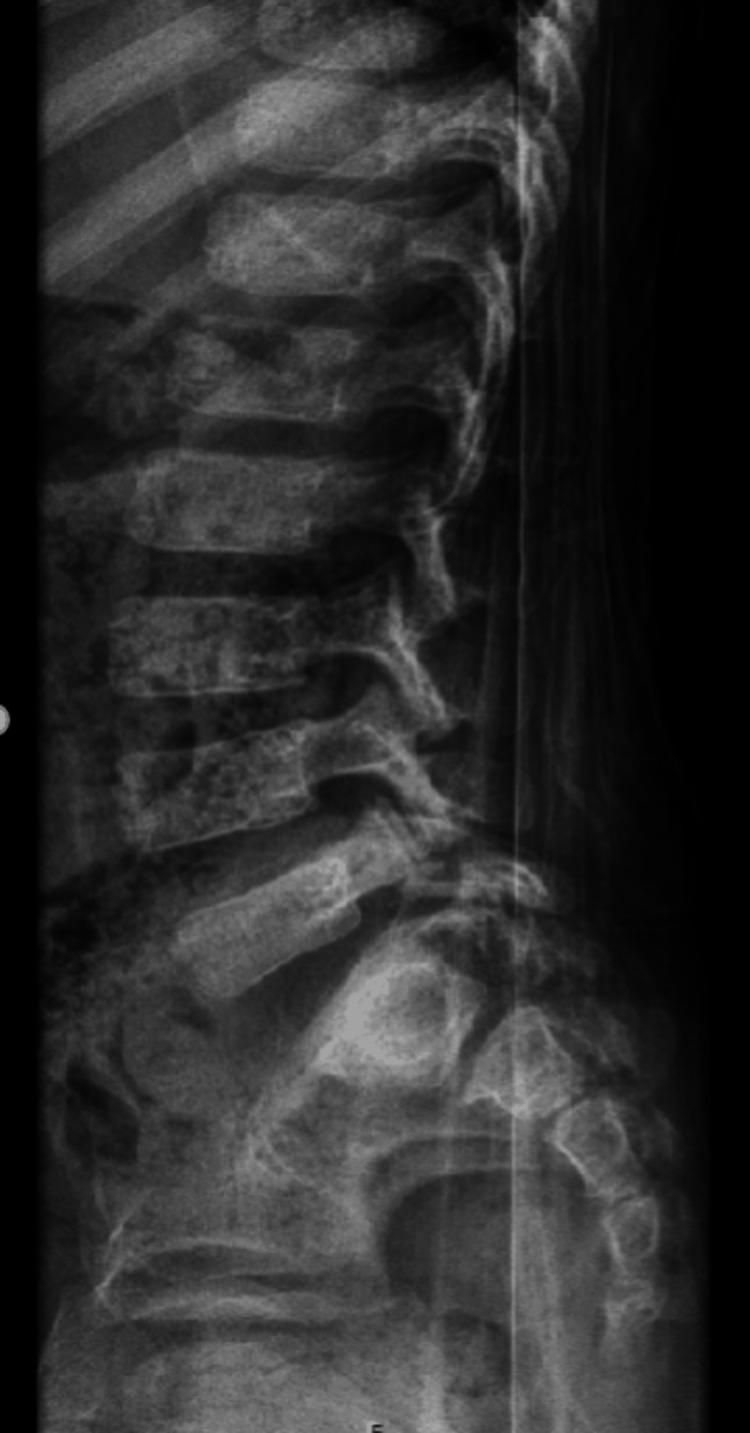
Radiograph showing suspected spondylolisthesis of L5-S1

AP, lateral, and oblique X-rays were taken, showing suspected spondylolisthesis of L5-S1 with L5 isthmic fractures and a sacral fracture (Figures 1-2). A computed tomography (CT) scan confirmed the presence of bilateral L5-S1 facet joint fracture-dislocation, classified as traumatic spondylolisthesis of L5-S1, type II-C according to Wiltse-Newman, grade I according to Meyerding, and as type IIIA according to Vialle et al. classification [1].

**Figure 3:**
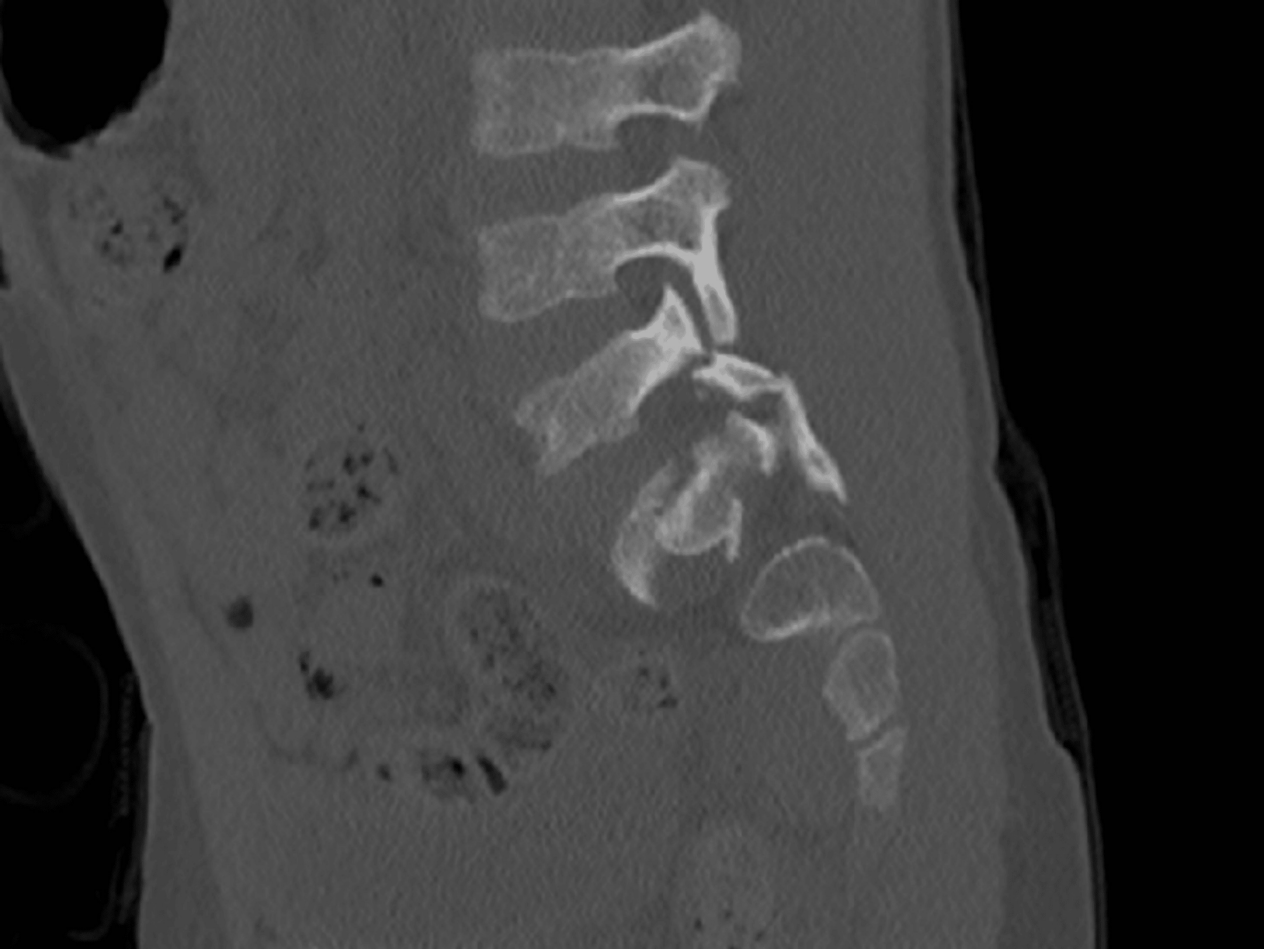
Left L5-S1 facet fracture-dislocation

**Figure 4:**
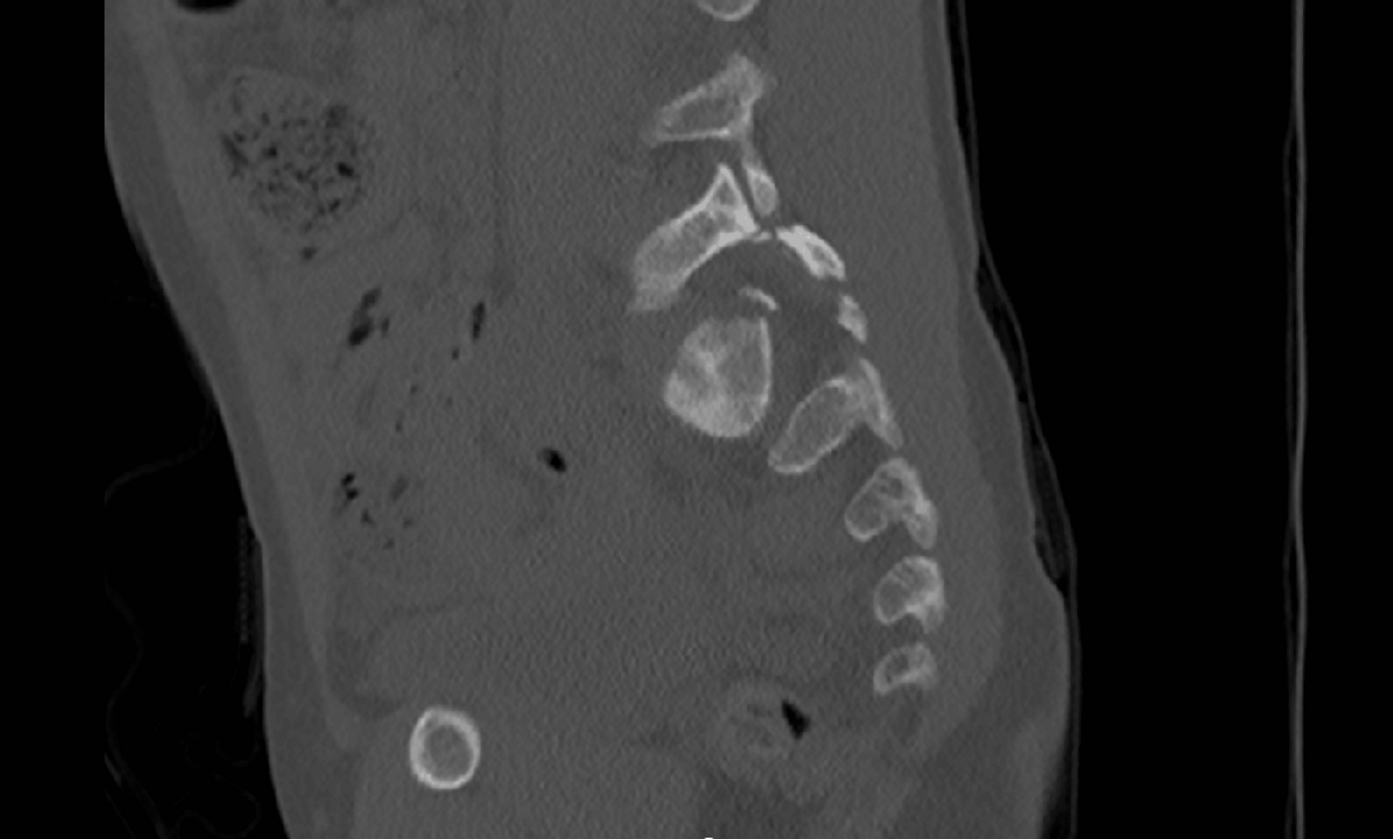
Right L5-S1 facet fracture-dislocation

**Figure 5:**
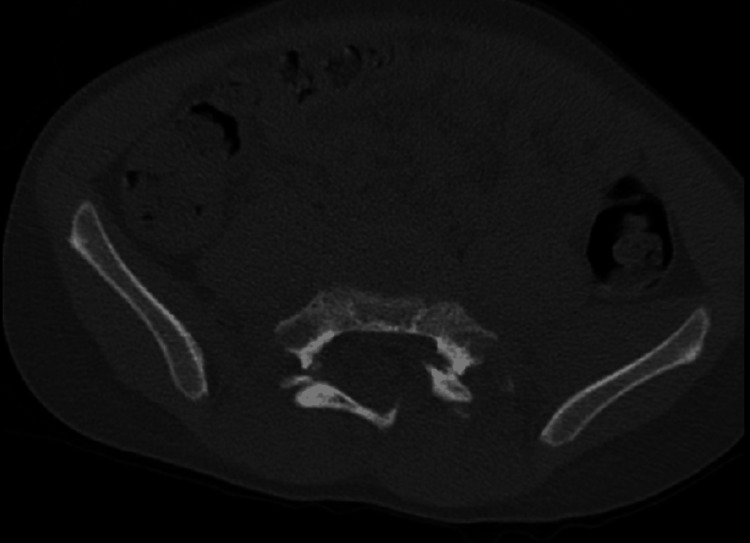
Sacral fracture intersecting the sacral wings at the S1 and a horizontal vertebral body fractures at S2 level, behaving as a spinopelvic dissociation

**Figure 6:**
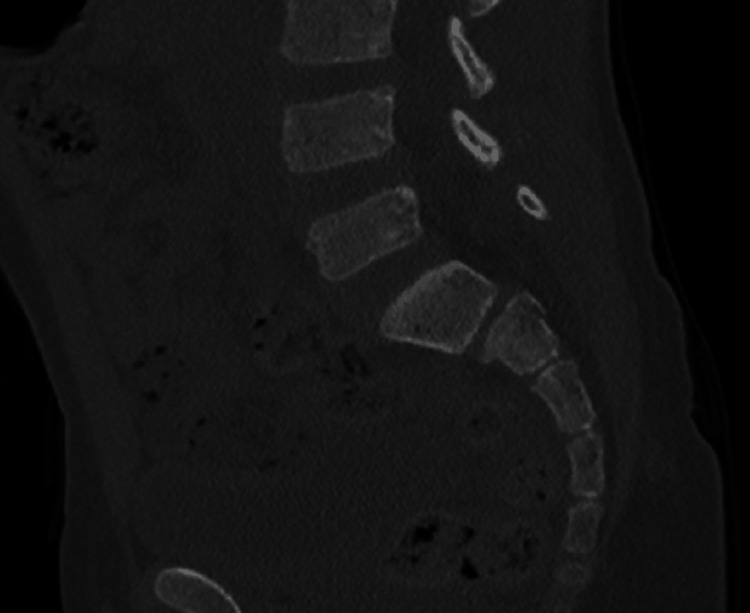
Sacral fracture intersecting the sacral wings at the S1 and a horizontal vertebral body fractures at S2 level, behaving as a spinopelvic dissociation

**Figure 7:**
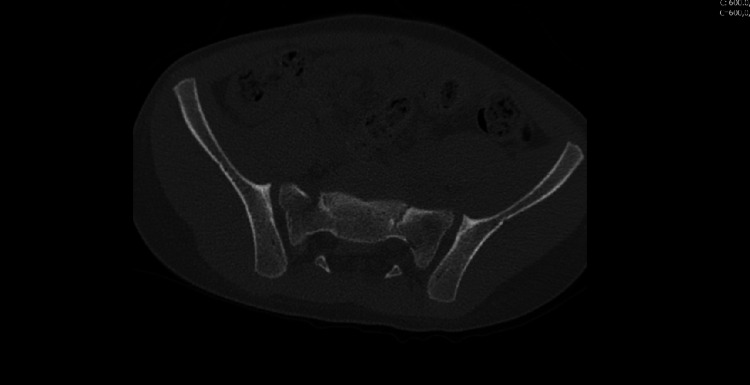
Sacral fracture intersecting the sacral wings at the S1 and a horizontal vertebral body fractures at S2 level, behaving as a spinopelvic dissociation

**Figure 8:**
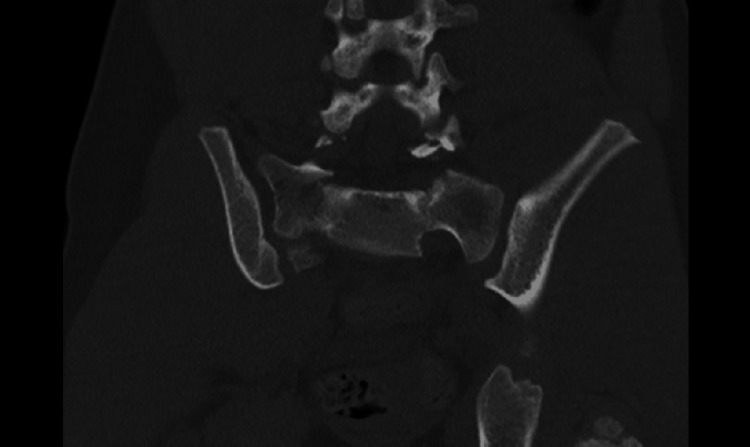
Sacral fracture intersecting the sacral wings at the S1 and a horizontal vertebral body fractures at S2 level, behaving as a spinopelvic dissociation

The CT images also showed a sacral fracture intersecting the sacral wings at the S1 level and horizontal vertebral body fractures at S2, manifesting as a spinopelvic dissociation (Figures 3-8).

**Figure 9:**
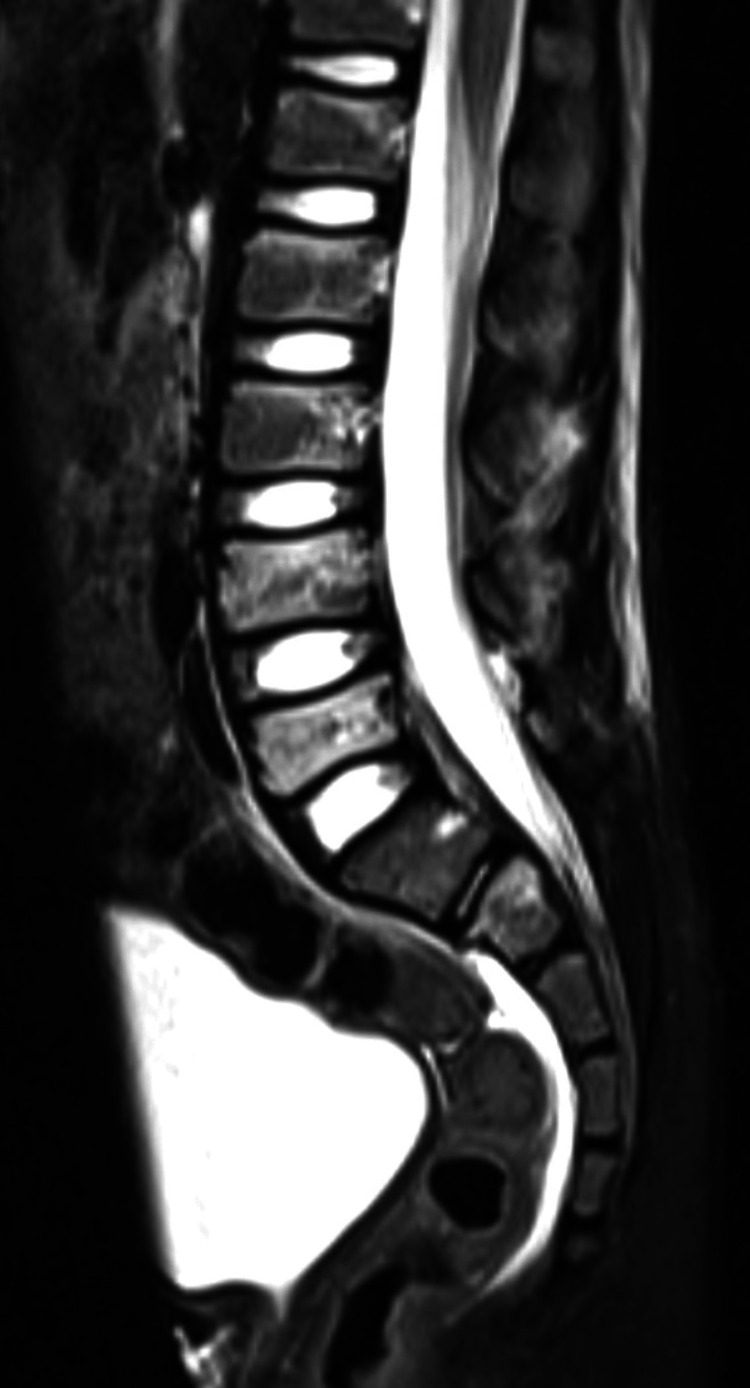
MRI showing injuries of the yellow ligaments and absence of disc injuries

**Figure 10:**
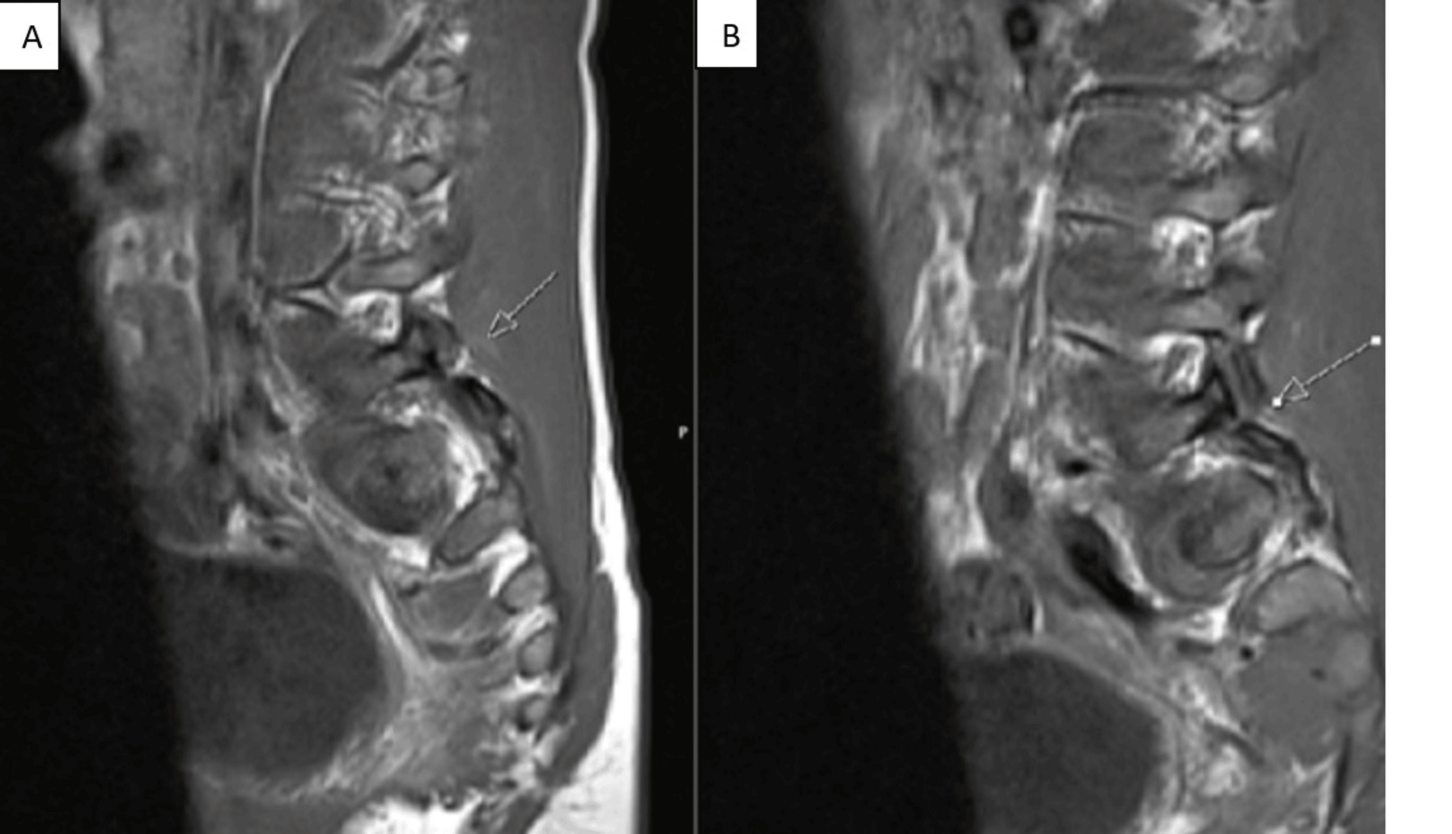
L5-S1 facet joint fracture-dislocation (A) Left L5-S1 facet joint fracture-dislocation. (B) Right L5-S1 facet joint fracture-dislocation

The patient underwent an MRI, revealing injuries of the yellow ligament and absence of disc injuries and edema in the fracture zone (Figures 9-10).

**Figure 11:**
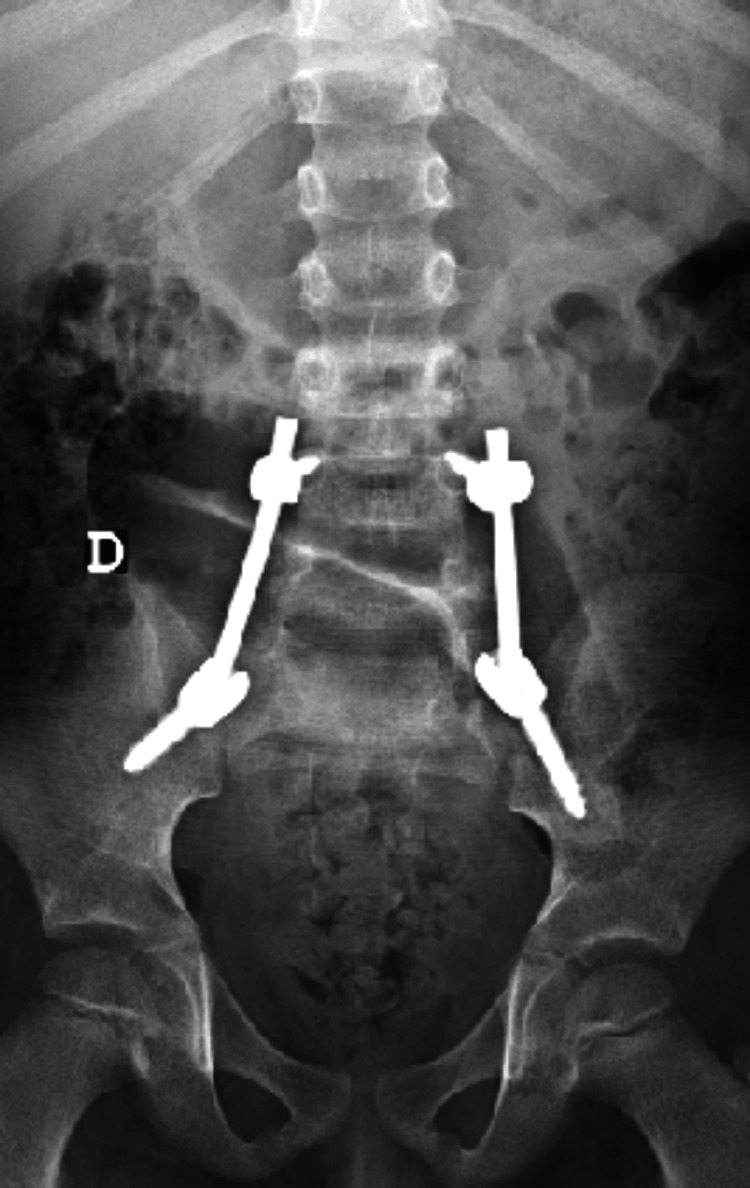
Postoperative radiograph

**Figure 12:**
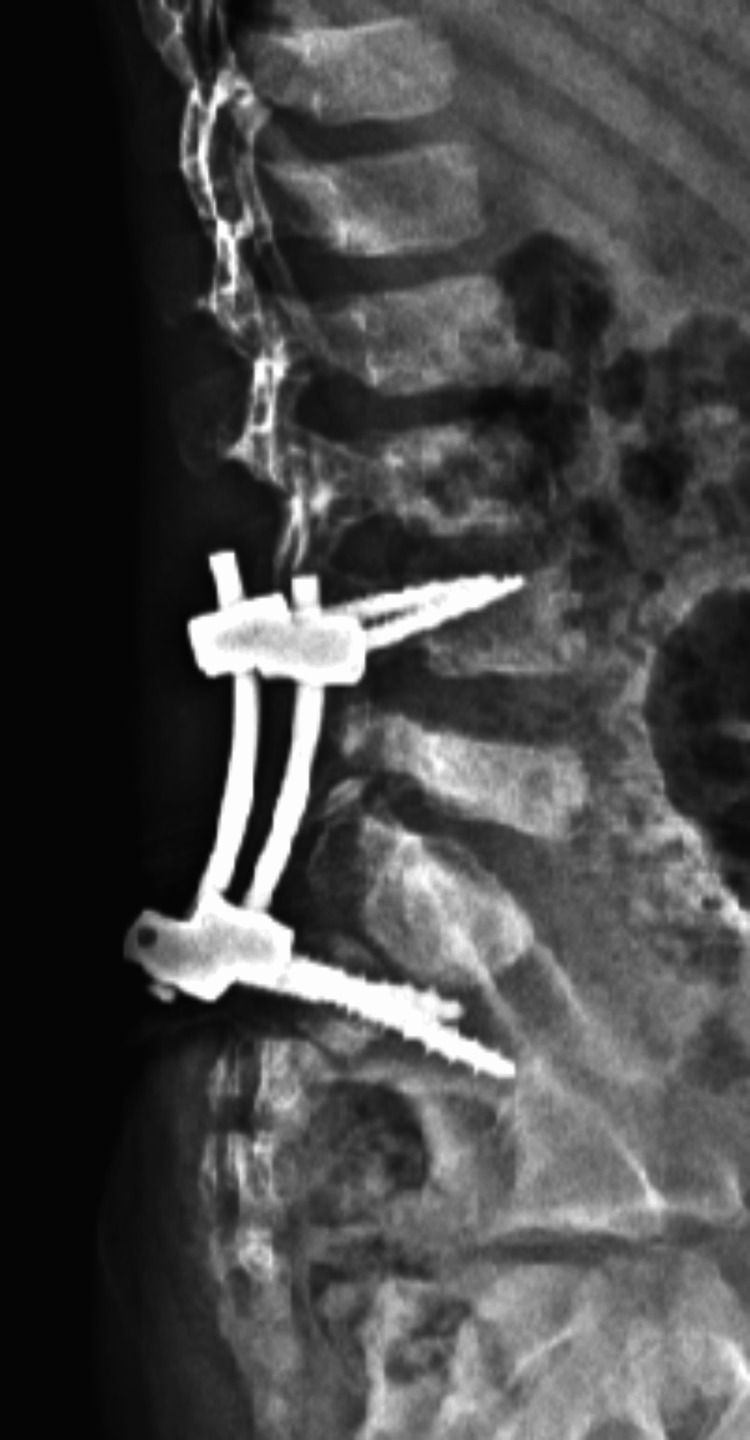
Postoperative radiograph

Despite the absence of neurological deficits, the presence of this traumatic spondylolisthesis with unilateral dislocation of the L5S1 facet and the association with this type of sacral fractures took us to perform an early surgical treatment with open reduction of the L5-S1 facet joint dislocation associated with posterior lumbopelvic fixation (L4-iliac) (Figures 11- 12).

**Figure 13:**
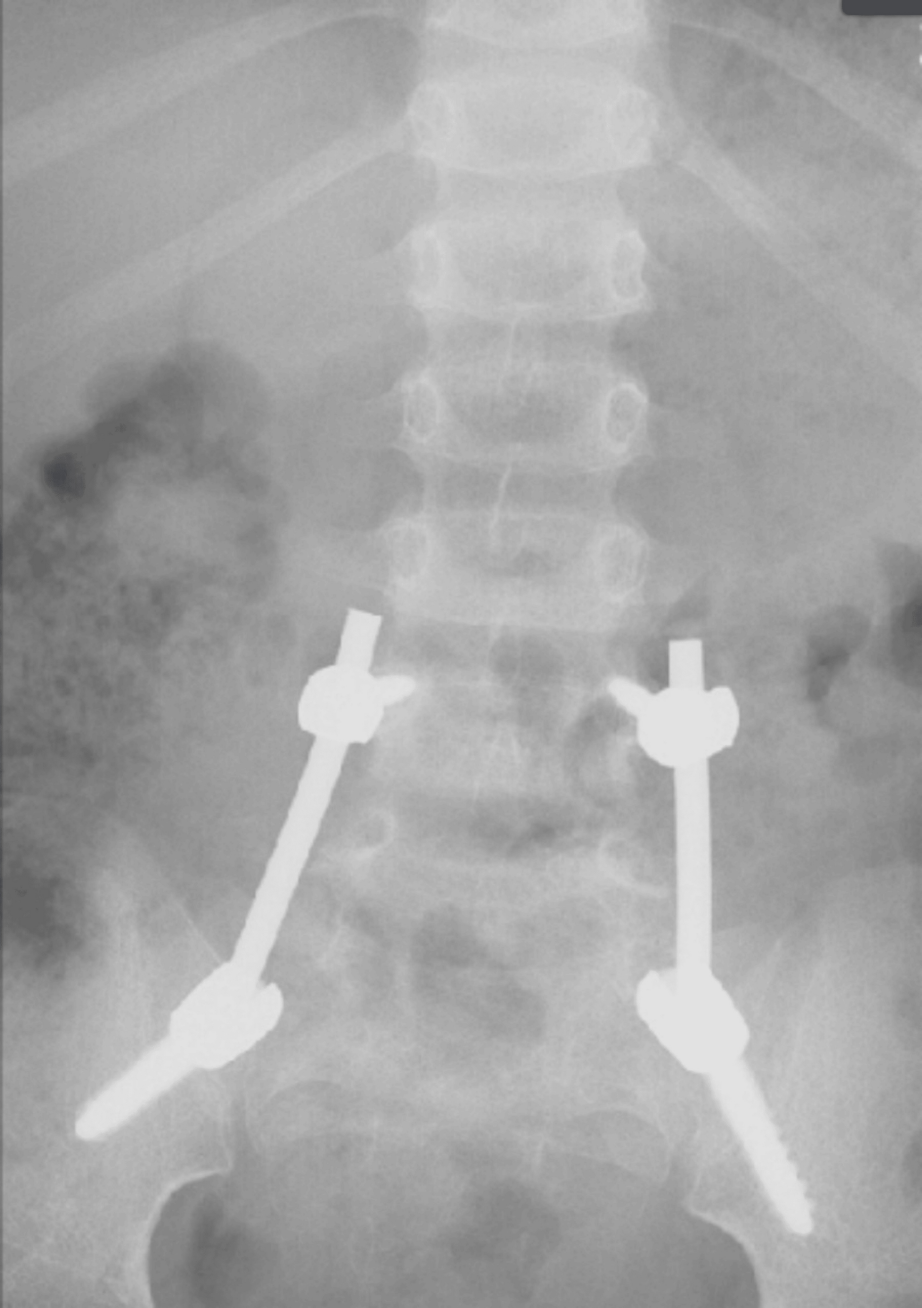
Radiograph at three months postoperatively showing good clinical progression

**Figure 14:**
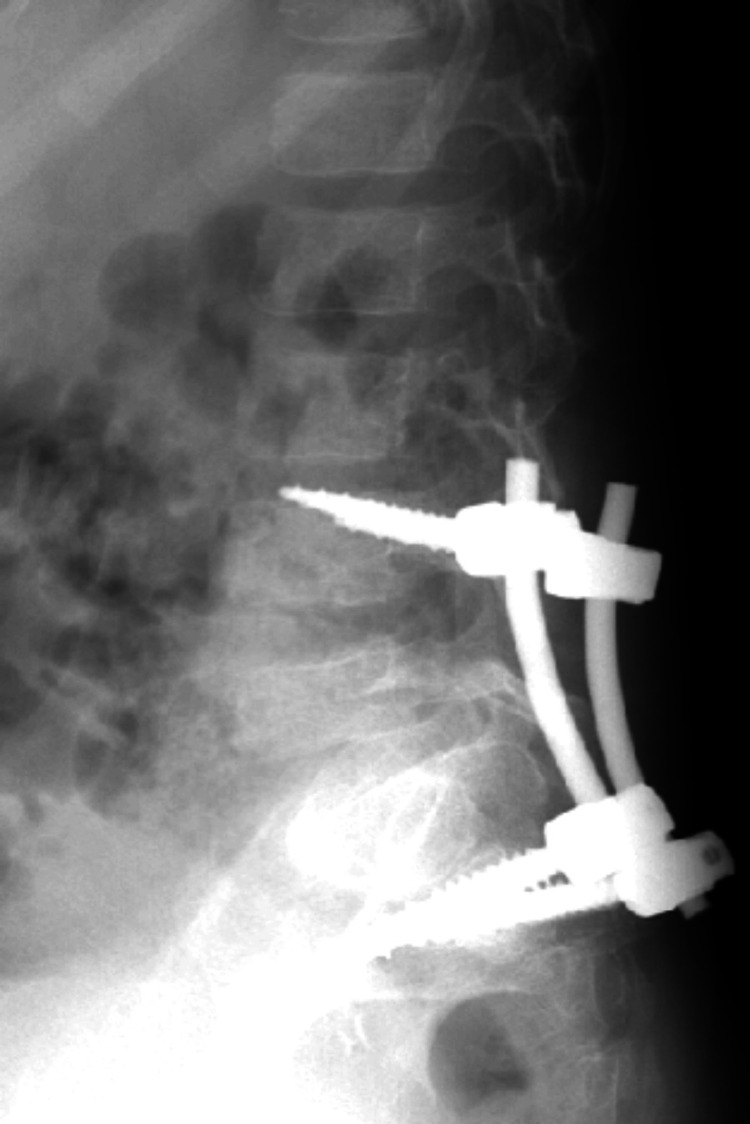
Radiograph at three months postoperatively showing good clinical progression

The postoperative period showed favorable scar evolution, with the patient able to stand and walk with crutches and was discharged three days after surgery. The patient had a good clinical progression with gradual recovery of mobility and independent walking. Radiographs at three months are presented (Figures 13-14).

**Figure 15:**
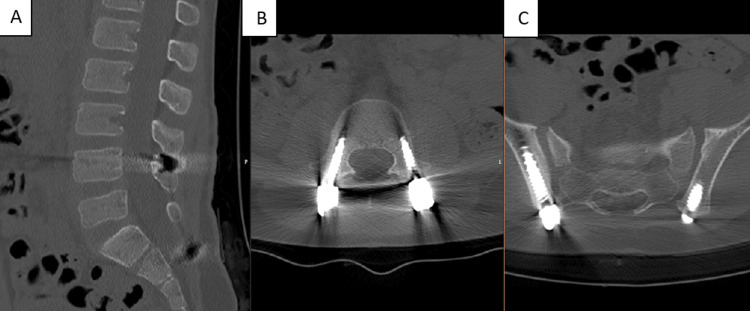
CT scan images at six months postoperatively (A) Sagittal view. (B) Axial view-L4 screws. (C) Axial view-iliac screws

**Figure 16:**
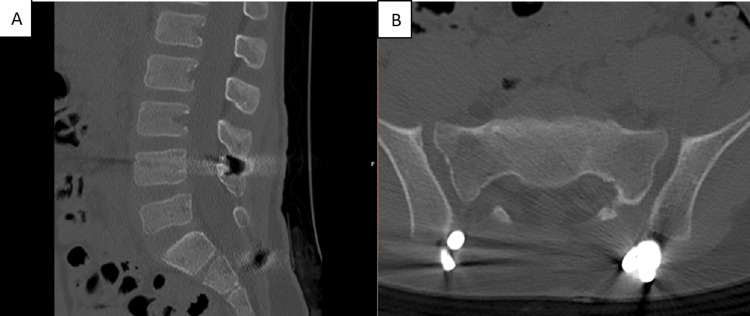
CT scan images at six months postoperatively (A) Sagittal view. (B) Axial view-iliac screws

**Figure 17:**
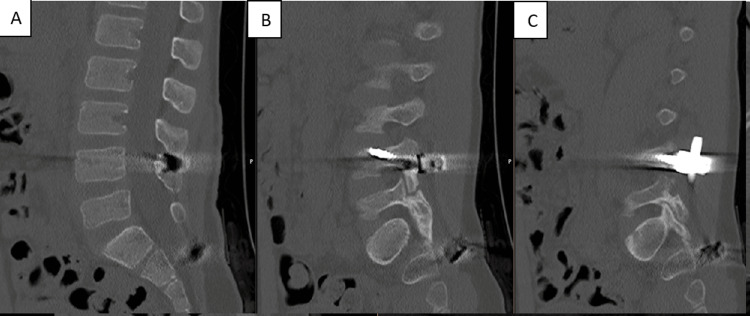
CT scan images at six months postoperatively (A) Sagittal view. (B)  Sagittal view-L4 screws. (C) Sagittal view-L4 screws

**Figure 18:**
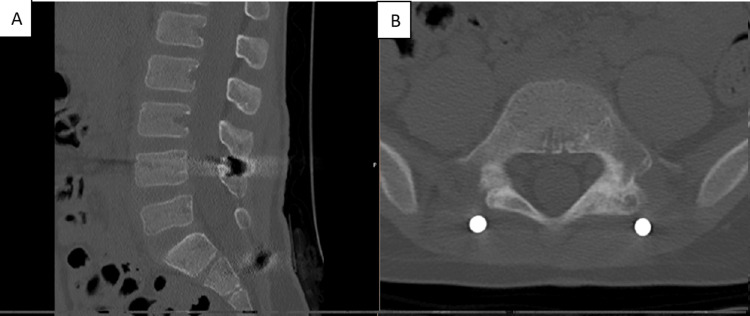
CT scan images at six months postoperatively (A) Sagittal view. (B) Axial view-iliac screws

CT images at six months postoperatively showed correction of the spondylolisthesis and consolidation of the L5-S1 facet joint fracture-dislocation and sacral fracture (Figures 15-18).

**Figure 19:**
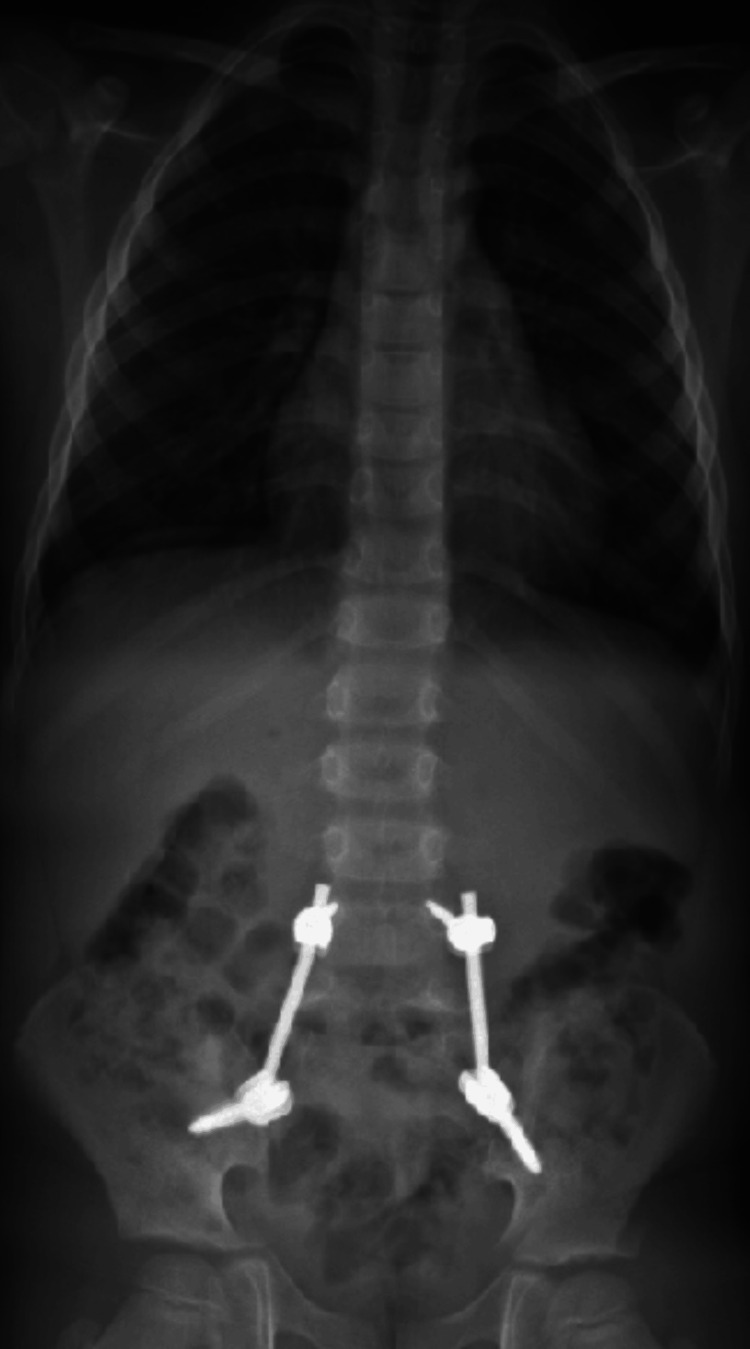
Radiograph at 12 months postoperatively

**Figure 20:**
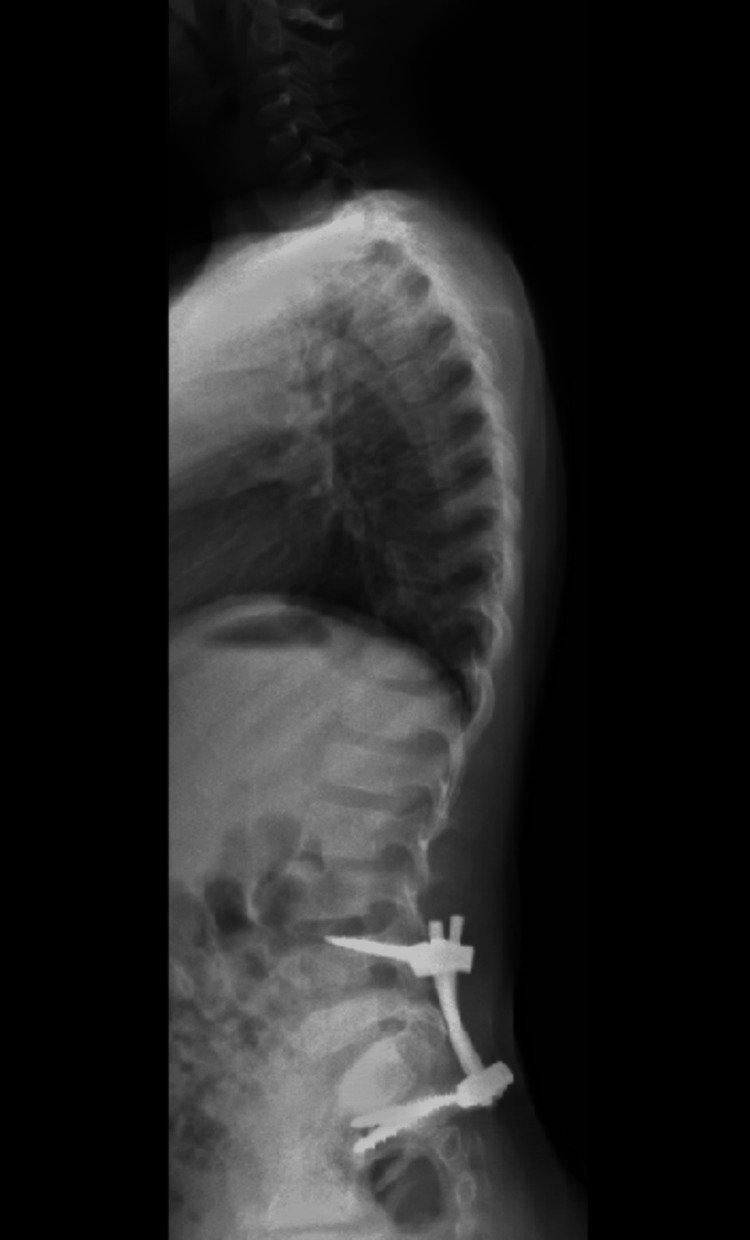
Radiograph at 12 months postoperatively

Radiographs at 12 months postoperatively are presented (Figures 19-20).

**Figure 21:**
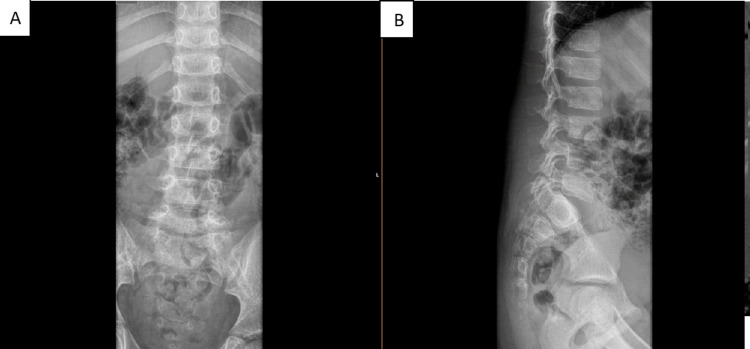
Radiograph after removal of the osteosynthesis material (A) Anteroposterior view. (B) Lateral view

One year later, the patient was clinically well, without any limitations, and underwent removal of the osteosynthesis material (Figure 21).

## Discussion

Most authors consider a combination of hyperflexion with compression as the commonest mechanism of injury to produce bilateral L5-S1 dislocation. The presence of a presumed unilateral rotatory element is implicated in unilateral facet dislocations [3].

Traumatic spondylolisthesis with lumbosacral facet dislocation is a rare injury, and in the pediatric population, it almost exclusively occurs in association with high-energy trauma. Children typically have more ligamentous laxity which accounts for why only 2%-3% of spinal fractures or dislocations occur in a pediatric population [4,5].

Unilateral facet dislocation in the lumbosacral spine is extremely rare due to the more vertical sagittal orientation of the facet joint and the presence of robust muscular and capsuloligamentous structures that provide substantial stability. These are unlikely to cause neurologic compromise. On the contrary, bilateral dislocations, especially those associated with a significant degree of spondylolisthesis, can produce neurologic deficit. The most affected nerve root is S1 and even with the reduction of dislocation, only partial recovery of the nerve function should be anticipated [3].

Radiologic diagnosis of this injury is dependent on good-quality initial radiographs. However, emergency room radiographs are frequently inadequate and can be easily misdiagnosed as normal [3]. The presence of transverse process fractures serves as a sentinel sign and should raise suspicion of a lumbosacral injury. A CT scan study of the lumbosacral junction needs to be performed; it provides a good visualization of bony structures and allows verification of the spinal canal diameter.

Magnetic resonance imaging (MRI) is useful when the patient is stable and without neurological compromise since it provides a useful preoperative evaluation of the presence of ligaments or disc injuries [1].

Our case is different from prior traumatic lumbosacral dislocations reported in that the patient is a child, and the mechanism of trauma was compression by a wardrobe fall on the child, with bilateral involvement and normal neurological examination. There are other pediatric cases, and the neurological status at admission might be a valid predictor of outcome. According to Vialle et al. classification, based on the injury patterns, we classify this lesion as type IIIA-bilateral fracture with anterior dislocation of L5 [1].

While anatomical reduction of deformity in the sagittal plane may not be completely achieved in all cases, stability must always be achieved. In acute dislocations, reduction is easily achieved with a transpedicular screw instrumentation, but spondylolisthesis may require adjacent level arthrodesis to control progression. Including L4 in the construct allows for longer rods, and not placing an L5 implant allows for an increase in the contact area of L5 pars fracture, promotes the healing of the fracture, and leads to a better correction of lordosis. The indication for additional interbody fusion remains controversial, especially in the pediatric age. Some authors argue that in moderate degrees (Meyerding I-II), if a severe disc injury exists, the interbody fusion can provide solid support to the anterior spine and reduce the impact of slipping forces through L5-S1. So like others, we recommend evaluating the L5-S1 disc through MRI as a criterion for anterior arthrodesis [1,6].

In our case, we opted for only transpedicular fixation L4 and distal fixation in the iliac, given the slight degree of listhesis and the maintenance of lumbar lordosis. The fact that in our case a fracture of the sacrum at the S1-S2 level reinforces the need to complete the instrumentation at the iliac level.

## Conclusions

In cases of traumatic spondylolisthesis with L5-S1 subluxation, we recommend careful deformity reduction and posterior fixation to establish sagittal balance. The instability caused by the injury and the high frequency of associated neurological injuries are obviously strong indications for surgical treatment.
